# Predicting cognitive functioning in early psychosis: factors supporting and limiting generalizability of connectome-based models

**DOI:** 10.1038/s44277-025-00032-1

**Published:** 2025-06-04

**Authors:** Alexandra G. O’Neill, Melissa Pax, Jourdan H. Parent, Jorge Sepulcre, Joan A. Camprodon, Stephanie Noble, Joshua L. Roffman, Hamdi Eryilmaz

**Affiliations:** 1 Department of Psychiatry, Massachusetts General Hospital and Harvard Medical School, Charlestown, MA USA; 2 Gordon Center for Medical Imaging, Department of Radiology, Massachusetts General Hospital and Harvard Medical School, Charlestown, MA USA; 3 Department of Radiology and Biomedical Imaging, Yale PET Center, Yale School of Medicine, Yale University, New Haven, CT USA; 4 Department of Psychology, Northeastern University, Boston, MA USA; 5 Department of Bioengineering, Northeastern University, Boston, MA USA; 6 Center for Cognitive and Brain Health, Northeastern University, Boston, MA USA

**Keywords:** Cognitive control, Working memory, Psychosis, Diagnostic markers

## Abstract

Cognitive impairment is a core, intractable aspect of psychotic disorders that impacts functional outcomes. Establishing reliable neural predictors of cognitive functioning at the individual level is an important goal of precision psychiatry and may accelerate personalized treatment development. Connectome-based predictive models (CPMs) have shown promise in identifying brain connectivity patterns that predict cognitive outcomes, however, such models do not produce accurate predictions for all individuals or groups, limiting their generalizability. Here, we used CPMs to identify brain network patterns predictive of cognitive functioning in patients with early psychosis and examined individual clinical and sociodemographic factors that may impact prediction accuracy. Leveraging the imaging data from the Human Connectome Project for Early Psychosis (HCP-EP; *N* = 93), we found that outcomes can be accurately predicted for general and fluid cognition. The generalizability of these models was assessed by predicting cognitive performance in an independent sample of patients (*N* = 20) with early psychosis, which revealed moderate accuracy but also sensitivity to the number of input features. Although predictive features were generally widespread, a virtual lesioning analysis showed that edges involving the default mode, retrosplenial and somatomotor networks contributed most to the prediction of individual differences in cognition. Finally, dissecting the causes of model failure suggested that sociodemographic and clinical factors that are stereotypically associated with cognitive ability in early psychosis contribute to misprediction particularly in participants who do not fit this stereotypical association. Our findings suggest that individual factors related to misprediction can inform and potentially improve predictive models of cognition in early psychosis.

## Lay summary

Cognitive difficulties in individuals with psychosis are common and hard to treat. Machine learning models that use MRI data to predict individual patients’ symptoms are increasingly available, however, these algorithms don’t produce accurate predictions for everyone. Our analysis of MRI data from recently diagnosed psychosis patients showed that MRI-based predictions of cognitive symptoms were more accurate for patients who had similar clinical and socioeconomic profiles and that models can potentially be improved considering these factors.

## Predicting cognitive functioning in early psychosis: factors supporting and limiting generalizability of connectome-based models

Take a Deep Dive into this article

## Introduction

Cognitive impairment is a core, debilitating aspect of psychotic disorders and has been linked to poor functional outcomes [1, 2]. Cognitive deficits are often present prior to the onset of psychotic symptoms [3] and youth at clinical high risk for psychosis exhibit attenuated deficits in various cognitive domains [4]. The early phase of psychosis is considered a critical period when treatments may have the greatest impact [5]. Since the cumulative effects of long-term medication use and chronicity is minimal in the early course of illness, it also presents an opportunity to identify robust, brain-based predictors of cognitive impairment largely free of confounding factors [6]. Identifying such predictors can potentially refine diagnostic features through patient subtypes and improve our understanding of individual differences in brain function and behavior.

Connectome-based predictive modeling (CPM) has shown substantial promise in identifying circuit-based predictors of outcomes in numerous neuropsychiatric disorders [7–9], as well as in transdiagnostic populations [10]. Nevertheless, models often fail to accurately predict clinical outcomes for many individuals [11, 12]. Recent work by Greene et al. has shown that model failure is systematic and suggested that connectomic predictive models do not represent unitary cognitive outcomes but rather reflect an interaction of clinical, cognitive, and sociodemographic factors.

The early phase of illness in psychotic disorders represents a period where substantial brain changes are likely to occur [13–15]. Therefore, various clinical factors (e.g., medication use, severity of psychotic symptoms) may influence the neural response to such changes and impact the ability of brain-based markers to predict cognitive outcomes. Therefore, it is crucial to identify key clinical and other biological factors that may influence the predictive power of a connectomic model in predicting cognitive outcomes in early psychosis. If a network identified by CPM predicts outcomes only for a subgroup of patients with psychosis, it would limit its generalizability. In addition, if success in prediction of outcomes depends on clinical or demographic factors, this may require developing models tailored to certain subgroups of patients.

To address these questions, in the current study we leveraged the neuroimaging data from the Human Connectome Project for Early Psychosis (HCP-EP) [6] and an independent local cohort to determine how well cognitive outcomes can be predicted from cortex-wide functional connectivity in early psychosis. Next, we identified the resting state networks that are important in predicting the cognitive outcomes. Finally, we assessed the prevalence of misprediction, identified several clinical, socioeconomic, and demographic variables that are associated with misprediction and investigated the differences in brain-behavior relationships in correctly and incorrectly predicted cases.

## Methods

### Participants

Two datasets were analyzed in the current study. For the main analyses of predictive modeling and misprediction, we utilized the publicly available HCP-EP dataset, whereas for the external validation of a model generated using the HCP-EP dataset, we used a cohort of patients with early psychosis enrolled in a neuroimaging study at Massachusetts General Hospital (MGH). For details on inclusion and exclusion criteria, as well as demographic and clinical characteristics of both samples, see Supplementary Methods and Table S1. HCP-EP is a multisite project that generated high quality neuroimaging, cognitive, clinical and genetic data in a cohort of individuals with early phase psychosis [6]. The August 2021 data release (https://www.humanconnectome.org/study/human-connectome-project-for-early-psychosis) involves 183 patients with early psychosis who had neuroimaging data. Among these participants, 93 had also behavioral data including the NIH toolbox cognition scores. One participant did not pass the head motion quality control (due to high ratio of above-threshold movements). Therefore, the final HCP-EP sample included 92 patients, whose data were used for the main analyses in the current study. The MGH sample included 20 patients with early psychosis. Two participants were excluded due to chance-level performance, leaving 18 participants for the external validation analysis.

### MRI acquisition and fMRI preprocessing

Both HCP-EP and MGH datasets underwent identical procedures for preprocessing and functional connectivity. For the details of MRI acquisition parameters, preprocessing, region of interest definitions, and computation of functional connectivity, see Supplementary Methods.

### Predictive modeling

As a first step, we used cortex-wide functional connectivity to predict 3 age-adjusted composite cognitive scores from the NIH toolbox [16–19], which include Fluid, Crystallized and Total Cognition. For details on these cognitive outcomes, see Supplementary Methods. For this analysis, we first created 100 unique train (70%) and test (30%) splits from the HCP-EP sample to cross-validate our predictive model on different test samples. See Fig. S1 for an illustration of the analysis pipeline. At each iteration, following established methods [20, 21], we first selected features using a feature-defining threshold (*p* < 0.01) for the participants in the training set. The selected features were those surviving this threshold for correlation between a particular edge (i.e., functional connectivity) and the behavioral outcome (e.g., Total Cognition score) across the participants in the training set. These edges were then divided into a positive and a negative set. The edges that were correlated positively with the cognitive outcome represented the positive feature set, whereas those that were negatively correlated with the outcome represented the negative set. To summarize the features, for each individual participant in the training set, we then summed the connectivity values across all thresholded features in the positive set, and subtracted from this the summed connectivity values across all thresholded features in the negative set, to arrive at a combined, individualized connectivity strength score. A linear model was then fitted to establish the relationship between the cognitive scores and the connectivity strength summary scores across the participants in the training set. Then, turning to participants in the test set, we generated predicted cognitive scores, based on the sum of their connectivity across the same set of thresholded features and the parameters of the trained model. At each iteration, prediction accuracy was calculated as the correlation (Pearson’s) between predicted and observed cognitive scores in the test set. We repeated this procedure for each of the 100 train/test splits and obtained 100 values of prediction accuracy for a given cognitive outcome. The significance of the predictions was determined using permutation testing, where the cognitive scores were randomly assigned to participants and the same cross-validation procedure described above was applied. This step was repeated 1000 times to obtain a null distribution of model performance. The *p*-value was computed as the proportion of permuted predictions numerically greater than the mean of the true prediction correlation (*p* < 0.05). The *p*-values were corrected for the three comparisons (i.e., cognitive outcomes) using Bonferroni correction. In addition to this train-test framework applied to the HCP-EP data, we also used an independent dataset acquired at MGH to externally validate our fluid cognition model constructed using the HCP-EP data. For details on external validation, a feature importance analysis, and analyses of alternative feature selection methods and site-specific models, please see Supplementary Methods.

### Misprediction analysis

To better understand why our models fail to predict cognitive scores for some individuals, we performed a misprediction analysis using a binary-outcome version of the predictive modeling framework explained above. To identify potential factors that might be associated with misprediction, we first quantified how often a participant was misclassified using misclassification index (MI) and examined whether this metric was related to a set of selected clinical and demographic covariates. These covariates included age, sex, race, parental socioeconomic status (SES), antipsychotic exposure (in months), Positive and Negative Syndrome Scale (PANSS) positive and negative symptom scores, and head motion during MRI. These covariates were selected as they reflect important demographic and clinically relevant features in early psychosis and were available in the HCP-EP dataset. For the details of this analysis, see Supplementary Methods.

## Results

### Connectome-based models predict cognitive outcomes in early psychosis

**Fig. 1:**
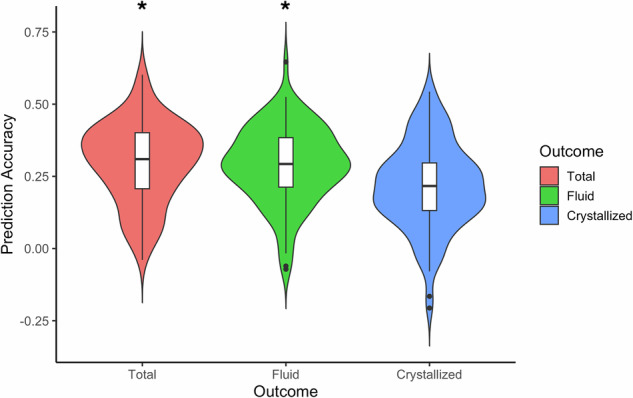
Prediction accuracy. Distribution of prediction accuracy over different cross-validation iterations is shown for three NIH toolbox outcomes. Permutation test revealed significant predictions for total and fluid cognition (*p* < 0.05 corrected). The box plot on each violin displays the interquartile range, with the center line representing the median prediction accuracy. The dots are outliers, and the asterisks (*) signify the significance of predictions for total and fluid cognition.

Predictive models based on connectivity strength summary scores were built using training sets and tested on previously unseen participants across 100 train-test splits. The prediction accuracy across these 100 iterations is illustrated in Fig. 1 for total, fluid and crystallized cognition. Mean prediction accuracy (r) was 0.30 for total (*p* < 0.05, corrected), 0.29 for fluid (*p* < 0.05, corrected), and 0.22 for crystallized cognition (*p* > 0.05, corrected). In exploratory analyses, we also assessed models using positive and negative summary scores separately and using a different feature selection threshold (*p* = 0.05). Models using combined scores generally led to numerically higher accuracy in prediction for all 3 outcomes than those using exclusively positive or negative features. Predictions for total and fluid cognition were also significant for the different feature selection threshold (*p* < 0.05), suggesting that these models are robust to changes in number of input features. See Supplementary Results for the impact of using more stringent feature thresholds (Table S2), as well as including subcortical features in the input correlation matrices on prediction accuracy (Fig. S3). To test whether our model generalizes to an independent dataset, we built a new model using all HCP-EP participants with fluid cognition as the outcome and used the parameters from this model along with its selected functional connectivity features to predict working memory performance (a core fluid cognitive ability) in the MGH sample (*N* = 18). The model using the combined feature set achieved numerically moderate but nonsignificant prediction accuracy in this external sample (r = 0.46, *p* = 0.056). However, this out-of-sample prediction appeared sensitive to the choice of feature selection threshold as more lenient threshold (*p* < 0.05) leading to a larger number of input features produced significant predictions (r = 0.48, *p* = 0.029), suggesting a degree of generalizability to other early psychosis populations and cognitive subdomains.

### Important network features for accurate predictions of cognitive functioning in psychosis

**Fig. 2:**
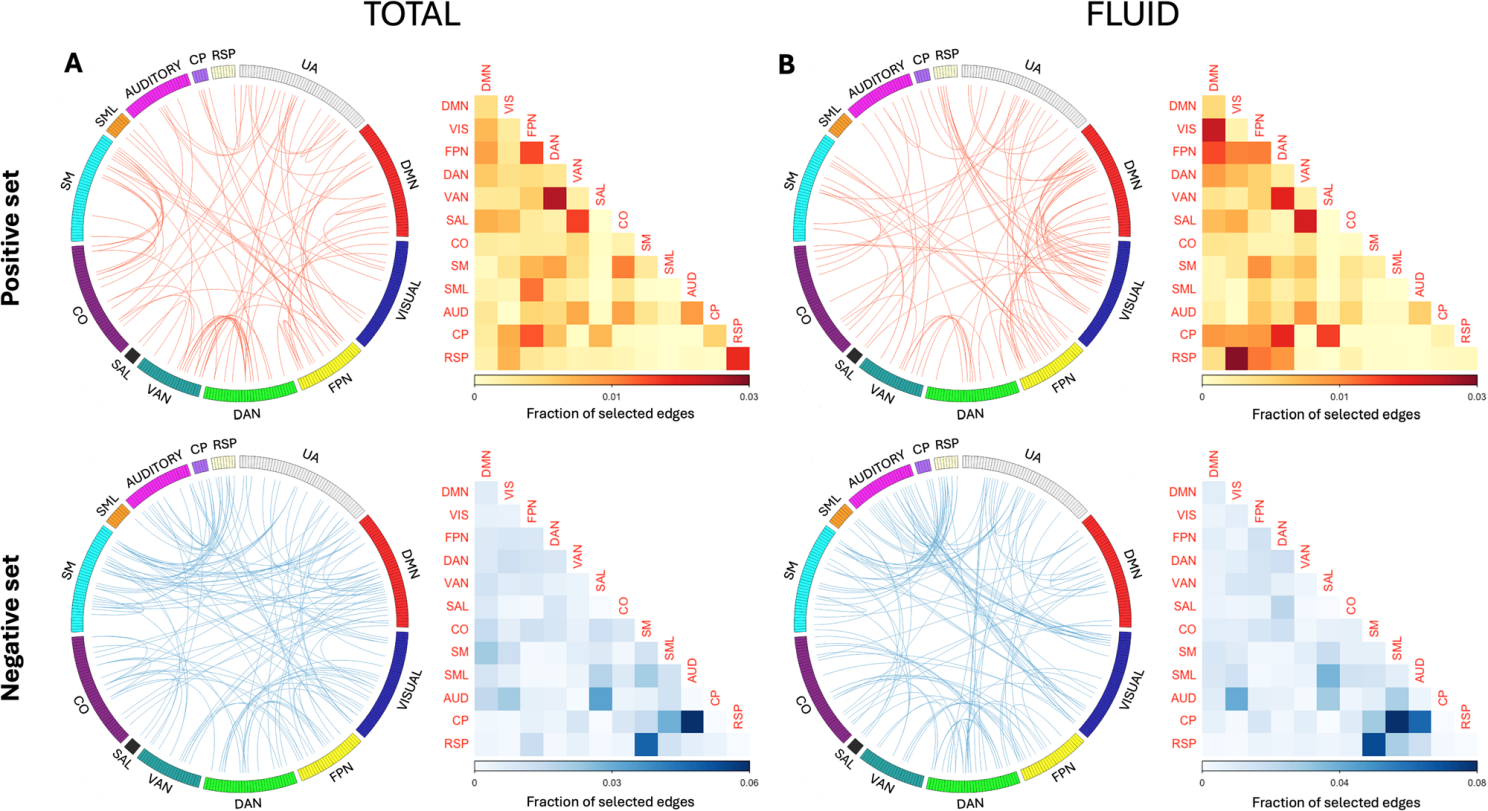
Important features. Network features that are important for predicting Total (**A**) and Fluid (**B**) Cognition scores are shown. Connectivity between the dorsal and ventral attention networks and between somatomotor and default mode/retrosplenial networks contributed the most to network summary scores for total cognition. Connectivity between the default mode and visual networks, between the dorsal and ventral attention networks, and between the retrosplenial and visual networks contributed most to network summary scores for fluid cognition. AUD auditory network, CO cingulo-opercular network, CP cingulo-parietal network, DAN dorsal attention network, DMN default mode network, FPN frontoparietal network, RSP retrosplenial network, SAL salience network, SM somatomotor network, SML lateral somatomotor network, VAN ventral attention network, VIS visual network.

Given models with significant predictive power for two of the main cognitive outcomes, we identified the features contributing to the network summary scores used in the respective predictive models, which are shown in circular plots in Fig. 2. Overall, edges included in the predictive model were widespread, although there was some degree of enrichment in particular networks. Among the edges positively correlated with total cognition, connectivity between dorsal and ventral attention networks appeared to disproportionately contribute to the summary score. Important features among the negatively correlated set included connectivity between somatomotor network and default mode/retrosplenial networks. For fluid cognition, connectivity between the default mode and visual networks, and dorsal-ventral attention network coupling were among the important features in the positive set. Among the features negatively correlated with fluid cognition, multisensory network coupling (e.g., visual-auditory) and somatomotor network connectivity with retrosplenial network appeared to have contributed most to the summary scores. The summary matrices depicting the average fraction of edges selected across different cross-validation iterations for each network are shown in Fig. 2. For completeness, the features predicting crystallized cognition is illustrated in Fig. S4.

**Fig. 3:**
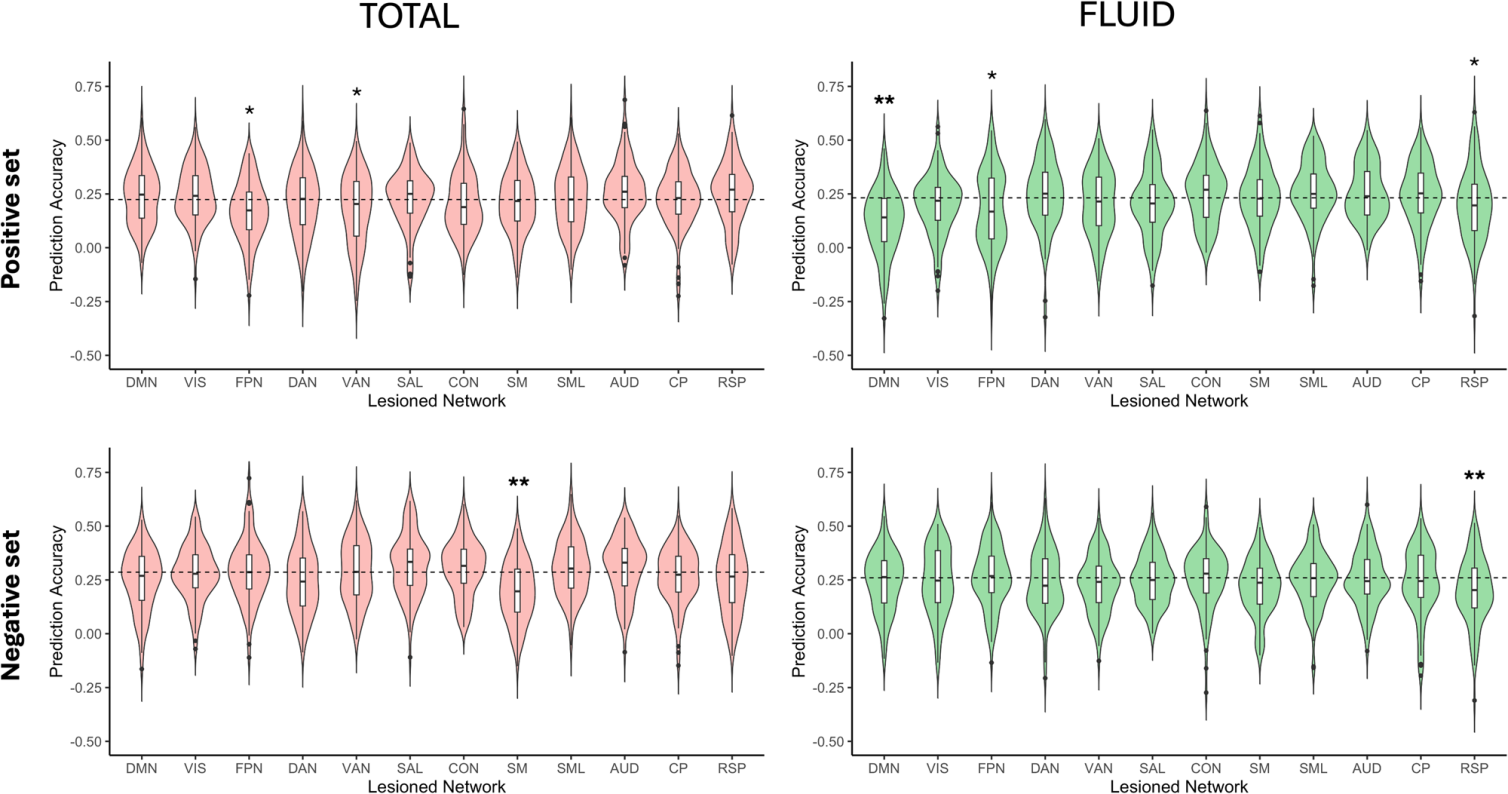
The effect of lesioned networks on prediction accuracy. The distribution of prediction accuracy after each network was virtually lesioned is shown for total and fluid cognition. The removal of the DMN from the positive feature set and of somatomotor and retrosplenial networks from the negative feature sets led to significant reductions in mean prediction accuracy for these outcomes. Values with single asterisks (*) depict nominally significant changes in prediction accuracy (*p* < 0.05). Boldfaced values with double asterisks (**) depict significant changes after correction for multiple comparisons (*p* < 0.05 corrected). The box on each violin plot displays the interquartile range, while the center line depicts the median and the dots represent the outliers. The dashed lines represent the original prediction accuracy values for the respective outcome/feature set when all networks were used in the model.

To quantify the importance of individual networks for the prediction of cognitive scores, we virtually lesioned all edges of each of the 12 Gordon networks (one network at a time) and reran the predictive models with the remaining edges. Fig. 3 shows the distribution of prediction accuracy across iterations after the removal of individual networks. For the prediction of total cognition using the negative feature set, lesioning the somatomotor network led to a significant decrease in prediction accuracy (mean/std error of change in prediction accuracy = −0.088/0.014, *p* < 0.05 corrected). When predicting fluid cognition using the positive feature set, lesioning the default mode network (DMN) significantly diminished the prediction accuracy (mean/std error of change in accuracy = −0.115/0.015, *p* < 0.05 corrected). For the prediction of fluid cognition using the negative set, lesioning the retrosplenial cortex led to a significant reduction in prediction accuracy (mean/std error of change in prediction accuracy = −0.060/0.015, *p* < 0.05 corrected). At uncorrected levels (*p* < 0.05), lesioning the fronto-parietal network worsened predictions for both total and fluid cognition (mean/std error of change in prediction accuracy = −0.055/0.014 and −0.053/0.017 respectively). Table S3 depicts the mean change in prediction accuracy after virtually lesioning each of the 12 networks for both cognitive outcomes.

### Characterizing misprediction

**Fig. 4:**
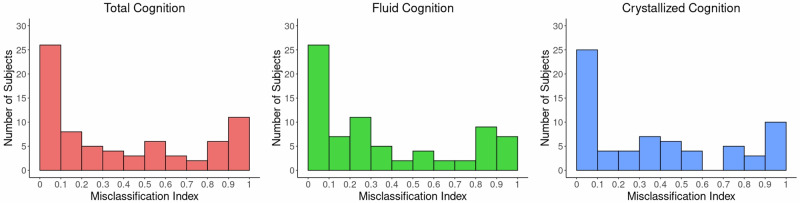
Misclassification. Histogram of misclassification index is shown for each NIH toolbox outcome. Most participants are classified correctly by the SVM for all three cognitive outcomes (MI < 0.5). However, a subset of participants are consistently misclassified over 100 iterations (MI > 0.5).

While connectomic models produced significant predictions of cognitive scores in early psychosis, the predictions consistently failed for a subset of patients in the HCP sample. We investigated misprediction using a SVM algorithm which generated binary predictions of the 3 composite NIH toolbox outcomes (i.e., low vs. high scorer). The model was run 100 times using random subsampling of training participants and tested on a left-out test participant in leave-one-out cross-validation. The distribution of MI is depicted in Fig. 4. A subset of participants was frequently misclassified in these predictions (38, 32, and 32% of all subjects had MI > 0.5 respectively for total, fluid and crystallized cognition).

**Fig. 5:**
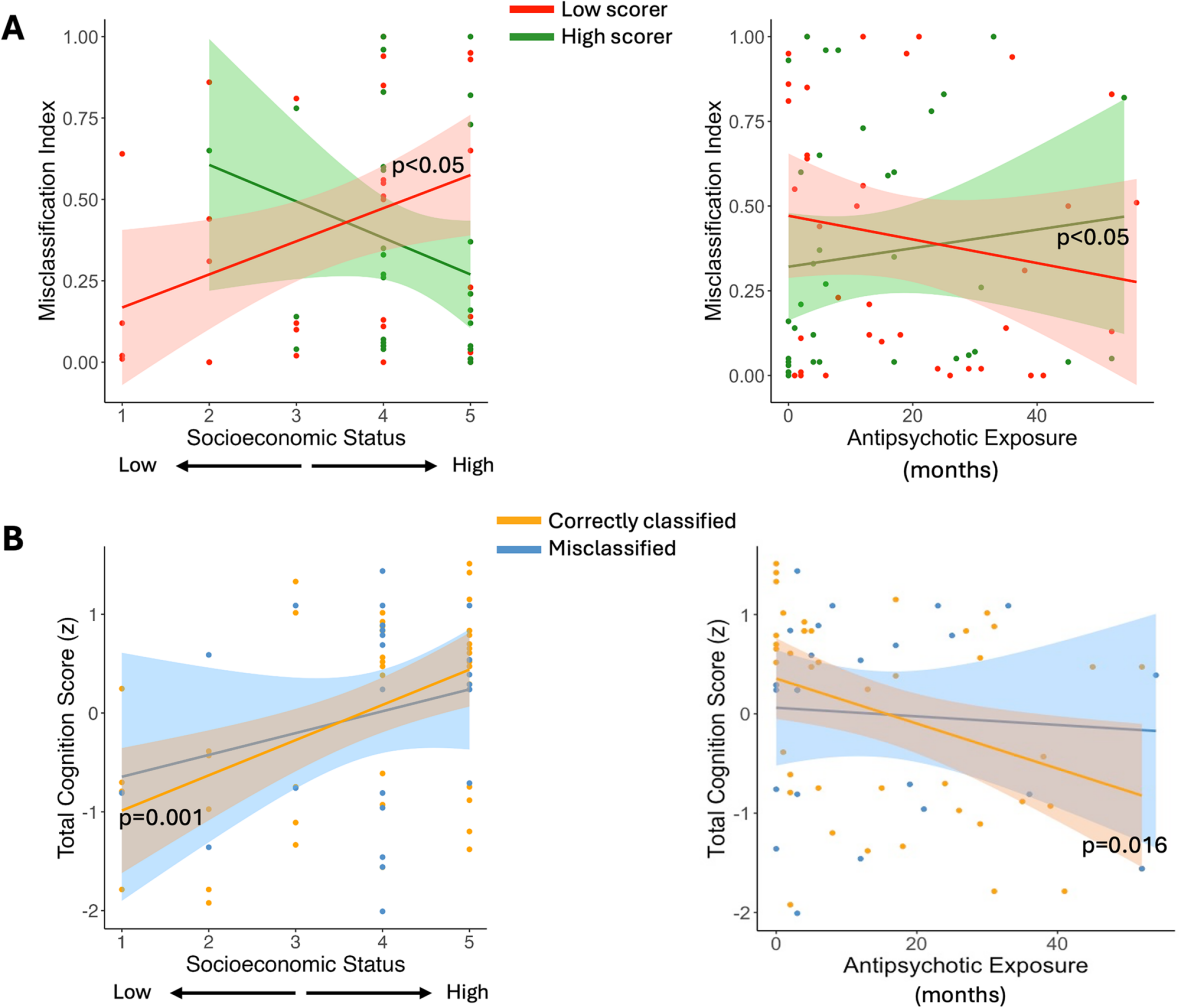
Relationships among misclassification index, covariates and cognitive outcomes. **A** The scatterplots show the relationships between misclassification index and parental SES and between misclassification index and duration of antipsychotic exposure for both low (red) and high (green) scorers. **B** The relationship between each covariate of interest and total cognition is shown for correctly and incorrectly classified participants. The significant correlations identified in correctly classified participants were either reduced or disappeared in misclassified participants. The significant associations are highlighted with their respective *p* values.

Next, we investigated whether participants with certain clinical and sociodemographic profiles were more likely to be misclassified. For this analysis, we considered 8 key clinical and demographic features that were available in the HCP-EP dataset. The results of this analysis are summarized in Fig. 5. Two of the covariates were marginally correlated with MI: SES among low-scorers on total cognition (r = 0.39, *p* = 0.017) and antipsychotic exposure among high-scorers on total cognition (r = 0.35, *p* = 0.033). A closer examination revealed a strong association between SES and total cognition scores in correctly classified participants (r = 0.48, *p* = 0.0012), whereas this association was not present in frequently misclassified participants (*p* = 0.27). Similarly, duration of antipsychotic exposure was negatively correlated to total cognition scores in correctly classified participants (r = −0.37, *p* = 0.016), whereas this correlation was not observed in frequently misclassified participants (*p* = 0.75). For the associations between MI and all 8 covariates, see Table S4. Finally, due to the strong relationship between the two identified covariates and cognitive scores in correctly classified participants, we tested the possibility that our models mainly predicted these covariates and not the cognitive outcomes. The prediction of total cognition remained significant (*p* < 0.05) after covarying for SES and for antipsychotic exposure in the HCP-EP sample, although prediction accuracy decreased from 0.30 to 0.26 and 0.28 respectively. Overall, the misprediction analysis suggests that the present models partially reflect factors that are stereotypically associated with cognitive ability, and points to ways these models may fail for individuals who do not fit this stereotypical association.

### Inverse models for misclassified participants

Finally, to examine the model differences between correctly and incorrectly classified participants, we built predictive models using one group and tested the model in the other. For this analysis, we built a new predictive model using only correctly classified participants (MI < 0.4). These models failed to predict cognitive scores in misclassified participants. The predictions were substantially misaligned especially for fluid cognition as the correlation between observed and predicted scores was negative (r = −0.29 for total, r = −0.62 for fluid, and r = −0.03 for crystallized cognition). This pattern suggested that network summary scores may be related differently to behavior in correctly and incorrectly classified participants. We then modified the low MI-trained model by inverting the sign of positive and negative network summary scores and retested this model in frequently misclassified participants. In this case, the predictions substantially improved (r = 0.28 for total, r = 0.62 for fluid, and r = 0.03 for crystallized cognition). Frequently selected features for this model, which exclusively used data from correctly classified participants are depicted in Fig. S5.

## Discussion

Using a data-driven approach, we investigated whether cognitive outcomes can be reliably predicted from functional connectivity in patients with early psychosis and examined important clinical and sociodemographic variables that may affect the prediction accuracy. We found that models successfully predicted fluid and general cognitive functioning in the HCP-EP sample, which was validated in an independent cohort of patients with early psychosis. Edges of several networks including the DMN, somatomotor network, retrosplenial network and FPN appeared to be important for accurate predictions. In addition, we found that the models failed to predict cognitive outcomes for some participants in the HCP-EP sample. A model failure analysis identified parental SES and duration of antipsychotic medication exposure as factors associated with misprediction and suggested that the prediction of fluid and general cognitive functioning can be improved for misclassified participants using inverted models. Taken together, our findings shed light on the utility of CPMs in predicting cognitive outcomes in early psychosis and highlight the importance of considering clinical and sociodemographic variables in predictive models of cognition in this population.

Cognitive impairment is a core feature of psychotic disorders [22]. Despite the significant impairment observed at the group level (e.g., effect sizes ranging from 0.64–1.2), patients with early psychosis exhibit considerable interindividual variability in cognitive functioning. Previous studies identified distinct patient subgroups in psychosis based on cognitive profiles [23–27]. Brain-based markers have also been used to account for differences between such subgroups. One study found differences in functional connectivity in the frontoparietal and motor networks between cognitively intact and impaired subgroups [28]. In the current study, we leveraged functional connectivity to predict cognition at the individual level without using cognitively defined subgroups. CPMs performed better when predicting fluid and total cognition compared to crystallized cognition. While total cognition represents the combination of the fluid and crystallized subdomains, our finding might reflect the impact of overlapping input features for fluid and total cognition outcomes selected during cross-validation on network summary scores. Furthermore, our fluid cognition model built using the HCP-EP sample predicted working memory performance in an independent sample of early psychosis patients. Although the significance of this external validation was sensitive to the choice of the feature-selection threshold, these results suggest that CPMs are generalizable to different early psychosis populations and arguably reflects the robustness of cross-validation in brain-behavior associations [29, 30]. The external validation of our HCP-EP model also supports CPMs’ reliability in predicting different subdomains of fluid cognition, which is consistent with the strong relationship between working memory and general cognition [31].

Our virtual lesioning analysis showed that removal of single networks does not generally lead to significant reductions in prediction accuracy. This suggests that the summary network score that the predictive model relies on is robust to certain changes in input data. There were, however, a few select networks that seemed to contribute more substantially to predictions. For instance, virtually lesioning DMN greatly reduced prediction accuracy for models predicting fluid cognition. Summary matrices showed that DMN’s connections with the visual network and FPN were predominantly selected during cross-validation. The role of the DMN in cognition has been demonstrated in numerous studies [32–34] and its dysfunction in psychotic disorders have been widely reported [35–37]. Consistent with these studies, our finding suggests that the DMN’s connectivity with the visual network and with the FPN support neural processes essential to fluid cognitive abilities in early psychosis. Another network whose removal greatly diminished prediction accuracy was the somatomotor network in models predicting total cognition. Specifically, coupling between somatomotor and retrosplenial networks was among the most selected features during cross-validation for this outcome. This finding is consistent with previous research that identified somatomotor network abnormalities as a transdiagnostic feature of cognitive dysfunction [38] and with the putative role of retrosplenial cortex in learning and memory [39]. Finally, virtually lesioning FPN reduced prediction accuracy in total and fluid cognition models. Summary matrices demonstrate that within-network FPN connectivity, as well as between-network FPN-DMN and FPN-SM edges were frequently selected in these models. FPN is thought to be essential to many executive functions including cognitive control [40, 41] and working memory [41]. Considering the putative central role of flexible FPN connectivity in numerous cognitive functions [42] and its disruption in psychosis [43], it is not surprising that its removal impacts prediction accuracy.

Our misprediction analysis demonstrated that predictive models consistently fail for a subset of patients with early psychosis. The patterns of relationships between MI and covariates such as SES suggest that the models learn a common profile of relationship between such covariates and the cognitive outcome and consequently fails for individuals that do not fit this profile, a pattern that was shown in seminal work investigating model failure on three large-scale imaging datasets [12]. We identified two covariates that have supported this phenomenon. For example, individuals with higher SES who scored high in cognitive outcomes were often correctly classified. However, those individuals who did not fit this stereotypical profile (high SES individuals with low scores and low SES individuals with high scores) were frequently misclassified. Parental SES has been identified as an important risk factor for developing psychosis [44, 45] (but also see [46]). SES can have implications for cognitive outcomes through a variety of associated factors including educational attainment [47], educational quality [48], and neighborhood disadvantage [49]. These factors (among numerous others) likely impact the brain networks that are predictive of cognition. Our partial correlation analysis suggests that SES explains a certain amount of variance in predicting cognition, however, the predictions based on summary connectivity scores were still significant after covarying for SES. Therefore, even though SES seems to be an important variable affecting the predictive power of connectomic models of cognition in early psychosis, such models also represent aspects of cognition that are not linked to SES. Our analysis also showed that the relationship between the network summary score and cognitive outcome was reversed in misclassified participants and therefore, simply using an inverted model dramatically increased prediction accuracy in total and fluid cognition models. This might suggest that the same networks that are predictive of cognition in the correctly classified group are also involved in the misclassified group, however, they are differently related to cognition. Alternatively, it is possible that the misclassified group exhibits a more idiosyncratic network structure underlying cognitive function, which leads to model failure.

Another covariate that showed a similar relationship with MI was duration of antipsychotic exposure. Correctly classified patients depicted a negative correlation between antipsychotic exposure and cognitive scores, whereas misclassified patients did not. To the extent that duration of antipsychotic exposure reflects duration of illness in the early psychosis population, declining trajectory of cognitive functioning after the first episode could explain this pattern among correctly classified patients. It is possible that the model relies on a neuromarker associated with the duration of antipsychotic exposure (or its effect on functional connectivity) to predict the cognitive outcome, in which case it might fail to predict outcomes for a subset of patients who do not depict a relationship between antipsychotic exposure and cognition. Together, our results suggest that predictive models learn complex profiles of relationships between the cognitive outcome and covariates that are relevant for the outcome. Given the implications for prediction accuracy, variables such as parental SES and antipsychotic exposure should be considered when studying individual differences in cognitive functioning in early psychosis.

Our study had several limitations. Since the HCP-EP dataset were collected at 3 different imaging sites, and due to the moderate sample size, our analysis plan was not optimal to capture site-related confounding factors. That said, the fact that all sites utilized the same scanner model, identical imaging sequences/protocols, and coordinated quality assurance tools helped minimize such potential confounds [6, 50]. Notably, after covarying for scanner site, the predictive power of our original models numerically diminished, however, the predictions remained significant. Therefore, while scanner site may explain some variance, its effects do not significantly alter model predictions. Additionally, site-specific models generalized well between the two major sites, whereas the predictions were less accurate in the smaller site, which might reflect the distinct characteristics of this cohort (e.g., higher proportion of individuals with affective psychosis). Moreover, data on several potentially relevant covariates including education and antipsychotic medication dose were missing for many HCP-EP participants in the Release 1.1, therefore, these variables were not included in the misprediction analysis. Additionally, we utilized a CPM algorithm involving a feature summarization step. It has been shown that other algorithms including regularized regressions can yield higher prediction performance for similar outcomes [51]. Using CPMs allowed us to directly parse features that contributed to predictions and therefore, facilitated evaluating feature importance [52].

In summary, we developed connectome-based models to predict cognitive functioning in early psychosis and identified brain network-level, demographic, and clinical factors that impact the accuracy of such predictions. Our findings highlight the importance of networks such as the DMN and somatomotor network, as well as SES and duration of antipsychotic use for accurate predictions. Future research can leverage misclassification index to identify patient subgroups and tailor predictive models accordingly. Finally, a similar misclassification framework can be adopted for models that aim to predict responses to cognitive therapies in early psychosis. These efforts can determine whether the clinical and demographic factors identified here are also implicated when predicting individual treatment responses.

### Citation diversity statement

The authors have attested that they made efforts to be mindful of diversity in selecting the citations used in this article.

## Supplementary information

Supplemental Material

## Data Availability

The raw imaging data from the Human Connectome Project for Early Psychosis are available at https://www.humanconnectome.org/study/human-connectome-project-for-early-psychosis. The imaging data for the MGH cohort are available from the corresponding author upon request. The scripts used in all data analyses in this manuscript are available here: https://github.com/heryilmaz/CogPred_HCP_EP.
