# Supplementary material for: Predicting cognitive functioning in early psychosis: factors supporting and limiting generalizability of connectome-based models
Source: NPP Digit Psychiatry Neurosci. 2025 Jun 4;3:11. doi: 10.1038/s44277-025-00032-1 (PMC12133584; doi:10.1038/s44277-025-00032-1)
Supplement: Supplementary file 1 — Supplemental Material [file 44277_2025_32_MOESM1_ESM.docx]

**Supplementary Information**

**Title:**

Predicting cognitive functioning in early psychosis: factors supporting and limiting generalizability of connectome-based models

**Authors:**

Alexandra G. O’Neill, BS^1^, Melissa Pax, BA^1^, Jourdan H. Parent, PhD^1^, Jorge Sepulcre, MD, PhD^2,3^, Joan A. Camprodon, MD, MPH, PhD^1^, Stephanie Noble, PhD^4,5,6^, Joshua L. Roffman, MD, MMSc^1^, Hamdi Eryilmaz, PhD^1^

**Affiliations:**

^1^Department of Psychiatry, Massachusetts General Hospital and Harvard Medical School, Charlestown, MA 02129

^2^Gordon Center for Medical Imaging, Department of Radiology, Massachusetts General Hospital and Harvard Medical School, Charlestown, MA 02129

^3^Department of Radiology and Biomedical Imaging, Yale PET Center, Yale School of Medicine, Yale University, New Haven, CT 06510

^4^Department of Psychology, Northeastern University, Boston, MA 02115

^5^Department of Bioengineering, Northeastern University, Boston, MA 02115

^6^Center for Cognitive and Brain Health, Northeastern University, Boston, MA 02115

**Supplementary Methods**

*Participants*

Two datasets were used in the current study: Human Connectome Project for Early Psychosis (HCP-EP) and Massachusetts General Hospital (MGH). HCP-EP is a multisite project that generated neuroimaging, cognitive, clinical and genetic data in a cohort of individuals with early phase psychosis as well as healthy controls [1]. The project focuses on the early phase of psychosis as this phase represents a critical period where neural plasticity is presumably higher and thus early interventions are more likely to be successful. Patients within 5 years of onset of psychotic symptoms were recruited across 4 sites in the United States (Indianapolis and Boston area). Enrolled participants were between 16-35 years of age, able to provide informed consent and were diagnosed with one of the following disorders based on DSM V definitions: schizophrenia, schizophreniform, schizoaffective disorder, psychosis not otherwise specified, delusional disorder, brief psychotic disorder, major depression with psychosis, or bipolar disorder with psychosis. Exclusion criteria included psychosis due to substance use or medical condition, IQ less than 70, history of HIV+ status, active medical condition known to affect cognitive functioning, contraindications to MRI, severe substance use in the 90 days prior to participation, electroconvulsive therapy in 12 months prior to participation, high risk for suicidal acts, and presence of overtly aggressive behavior. The MGH sample, which was used for external validation included 20 patients with early psychosis. The diagnostic inclusion/exclusion criteria for this sample largely mirrored those in the HCP-EP sample with the exception that the MGH sample did not include major depression with psychosis. Patients in the MGH sample were between 18-35 years of age, within 10 years of psychosis onset, able to provide informed consent, were fluent in English and were diagnosed with one of the following disorders based on DSM-V criteria: schizophrenia, schizoaffective disorder, schizophreniform disorder, psychosis not otherwise specified, delusional disorder, or bipolar disorder with psychosis. MRI incompatibility (including pregnancy), severe substance abuse within three months of participation, and unstable medical or neurological illness were exclusionary for this study.

*MRI acquisition*

For the HCP-EP sample, MRI data were acquired in 3T Siemens Magnetom Prisma Systems across 3 imaging sites. T1 (TR/TE = 2400 ms/2.22 ms) and T2 (3200 ms/563 ms) images were acquired at a voxel size of 0.8x0.8x0.8 mm^3^. Resting state fMRI images were collected using the following scan parameters: TR/TE/flip angle = 800 ms/37 ms/ 52^o^, in-plane resolution = 2 mm x 2 mm, slice thickness = 2 mm. Two sessions of 6-minute resting state scans were acquired with anterior-to-posterior and posterior-to-anterior phase encoding directions. Participants were asked to remain still during scanning and deformable foam cushioning was used to stabilize the head. Because the imaging data in the HCP-EP were acquired in 3 different scanner sites, multiple measures were taken to harmonize the data and scanner parameters, which included using Siemens-specific quality assurance tools and Functional Biomedical Informatics Research Network (FBIRN) phantoms. Benchmark measurements of scanner performance were taken using the FBIRN phantoms and inter- and intra-site variability were assessed. Additionally, three human participants traveled between the sites and were scanned in the respective scanners, which provided real-time assessments of scanner stability. If variance changes were noted, the sites worked with service engineers to correct the source of the change [2]. For the MGH sample, MRI data were collected in a 3T Siemens Skyra System. Structural image acquisition used the following parameters: TR/TE= 2530 ms/1.92 ms for the T1 (voxel size=0.8x0.8x0.8 mm^3^) and TR/TE= 3200 ms/406 ms for the T2 scan (voxel size=1x1x1 mm^3^). Resting state scans were collected with the following parameters: TR/TE/flip angle = 865 ms/39.4 ms/ 52^o^, in-plane resolution = 2.3 mm x 2.3 mm, slice thickness = 2 mm. A single 7-minute run with anterior-to-posterior phase encoding direction was acquired.

*fMRI preprocessing and functional connectivity*

Both datasets underwent identical procedures for preprocessing and computation of functional connectivity. Preprocessing was performed using FMRIB Software Library (FSL, version 5.0.7, https://fsl.fmrib.ox.ac.uk/fsl/fslwiki/FSL67), and Matlab (https://www.mathworks.com/products/matlab.html). The T1-weighted image was first reoriented to the anterior commissure–posterior commissure plane and segmented into gray matter, white matter, and cerebrospinal fluid. The anatomical image was then normalized to the Montreal Neurological Institute brain template (MNI152). The functional images were realigned to the middle functional volume, and head motion correction was performed using a six-parameter rigid body linear transformation, along with intensity normalization. Next, we performed a quality control analysis for head motion and scrubbed volumes with above-threshold motion [3]. One participant was removed due to excessive head motion (see Quality control analysis for head motion). Artifactual signals were removed via regression by applying a 12-parameter model (6 parameters from rigid body linear transformation and their temporal derivatives) and applying the component-based CompCor (5 parameters from cerebrospinal fluid signal and 5 parameters from the white matter signal). The residual volumes were used to compute functional connectivity. All fMRI data were normalized to the MNI152 brain template (3 mm^3^ isotropic), smoothed with a 6 mm full-width-at-half maximum Gaussian kernel and band-pass filtered to retain BOLD signal between 0.01 Hz and 0.08 Hz.

Regions of interest (ROIs) were defined using Gordon et al. parcellation, which includes 333 cortical parcels generated using surface-based boundary mapping [4]. These parcels are assigned to 12 canonical networks: default mode (DMN), visual (VN), frontoparietal (FP), dorsal attention (DAN), ventral attention (VAN), salience (SN), cingulo opercular (CO), somatomotor (SM), somatomotor lateral (SML), auditory (AN), cingulo parietal (CP), and retrosplenial (RSP). In this parcellation, groups with small number of parcels (<5) are not assigned to a separate network and named ‘unassigned’. Functional connectivity was computed as the Pearson’s correlation between each parcel’s average signal time course and every other parcel’s mean time course. 333x333 correlation matrices were converted to z-maps using Fisher’s z transformation, to enhance the normality of the distribution of correlations.

*Quality control analysis for head motion*

Given the influence of head motion on functional connectivity measures [3,5], we conducted a quality control analysis using Art Repair (https://www.nitrc.org/projects/art_repair/) and customized scripts [6]. Framewise displacement (FD) was computed at each time point. The participants with average FD greater than 0.5 mm (over the entire fMRI run) were not included in the analysis. In addition, volumes with FD > 0.5 mm were scrubbed from the connectivity analysis (0.9% of all volumes). Participants who had more than 10% of data loss due to scrubbing were removed from the analysis (1 participant was removed based on this criterion).

*Cognitive outcomes*

For predictive modeling analyses in the HCP-EP sample, age-adjusted cognition composite scores from the NIH Toolbox Cognition Battery were used as the main cognitive outcomes, which include Fluid Cognition, Crystallized Cognition and Total Cognition scores (variable names in the HCP-EP data sheets: *nih_fluidcogcomp_ageadjusted*, *nih_crycogcomp_ageadjusted*, and *nih_totalcogcomp_ageadjusted* respectively). The NIH Toolbox battery involves tests on executive function, attention, episodic memory, and language, and has been validated in various populations including individuals with psychosis [7-10]. In the Cognition module, Fluid composite score represents the individual scores from cognitive domains including working memory, episodic memory, inhibition, cognitive flexibility and processing speed, whereas Crystallized composite score represents scores from language domains such as oral reading and vocabulary. Finally, the Total Cognition score reflects the aggregate sum of Fluid and Crystallized component scores. In our analyses, we utilized each of the three composite scores as an outcome variable in the constructed predictive models.

*External Validation*

In order to test the generalizability of the global cognition models, we constructed one final model using all participants in the HCP-EP sample and tested this model in an independent replication sample, which included a local cohort of patients with early psychosis (N=20). In this local sample, the global cognition measures from the NIH toolbox were not available. However, all participants completed a working memory task. Therefore, in this analysis, we aimed to determine how well a global fluid cognition model in a large dataset generalizes to a narrow fluid cognitive domain (e.g., working memory) in a smaller sample. As working memory is considered a fundamental component of fluid cognitive abilities, we reasoned that the model would capture the features supporting working memory as well. The patients in the local sample recently underwent fMRI scanning at MGH as part of an imaging study, where their resting state scans and working memory performance data were acquired. The predictive model was constructed using the full HCP-EP sample following the procedures described in the Methods. The NIH toolbox cognition scores in the HCP-EP sample and working memory accuracy scores in the MGH sample were converted into standardized z-scores to allow for direct comparison between the different measures. The summary connectivity strength scores were calculated for the subjects in the validation set and the parameters from the HCP-EP-trained model were applied to obtain predictions for cognitive performance in the MGH sample. The participants in this replication sample performed a version of the Sternberg Item Recognition Paradigm, which assessed working memory function at 3 distinct memory loads. Briefly, participants were asked to memorize a set of 3, 5, or 7 letters. After a brief delay, they were presented with probe letters (one at a time) and indicated, using a keypad, whether the probe letter was one of the letters to memorize (target) or not (foil). Average working memory accuracy was used as the cognitive outcome to be predicted. Two participants were removed due to their chance-level performance (50% and 47%), leaving 18 participants in the external validation set. The prediction accuracy was defined as the Pearson’s correlation between predicted and observed working memory performance and the significance of the predictions was determined using the permutation procedure described in the Methods (N=1000).

*Feature importance*

We characterized the relative importance of each of the 12 canonical networks in predicting cognitive outcomes, by computing summary matrices indicating the prevalence of network features selected across different iterations of cross-validation. First, we calculated the number of edges selected per network at each of the 100 train-test splits. A summary matrix then represented the average fraction of a given network’s features (i.e., that network's importance) that were selected across iterations. In addition to the summary matrix visualization, to assess the importance of each individual canonical network for the predictions, we utilized virtual lesioning [11]. As part of this analysis, we first removed all within- and between-network edges of a given network and reran the models to predict cognitive scores in the HCP-EP cohort. As in the original analysis, model performance was determined using the correlation between observed and predicted outcomes. The change in prediction accuracy represented how important the lesioned network was for the prediction of the outcome. To determine the significance of network importance, we used a permutation procedure (n=500 due to extensive runtime) in which we repeated the virtual lesioning step by randomly removing M edges at each repetition and recalculating the prediction accuracy, where M was equal to the number of edges lost when virtually lesioning the network in question. This analysis was performed for each of the 12 canonical networks and separately for positive and negative feature sets for the two outcomes that produced significant predictions (i.e., total and fluid cognition). The p-value for a network was defined as the proportion of repetitions where randomly removing M edges from the whole brain resulted in a numerically greater decrease in prediction accuracy than when the network was virtually lesioned. The obtained p-values were corrected for multiple comparisons for 12 lesioned networks using False Discovery Rate correction (p<0.05).

*Impact of alternative feature selection methods on prediction accuracy*

All our main findings in the current study are reported at the feature-selection threshold of p<0.01. Given the arbitrary nature of this threshold, we tested a range of thresholds between p<0.0001 and p<0.05 (0.0001, 0.0005, 0.001, 0.005, 0.01, 0.05) to examine their impact on prediction accuracy for the total cognition outcome. We generated connectome-based models using these thresholds and cross-validated in 100 train-test splits in the HCP-EP dataset. These thresholds determined the input features used to calculate the connectivity strength summary score and the rest of the predictive modeling analysis remained identical. At each cross-validation iteration, prediction accuracy was calculated as the Pearson’s correlation between predicted and observed cognitive outcomes. The average prediction accuracy across 100 iterations determined the prediction accuracy at a given threshold.

All our main analyses utilized Gordon 333 parcellation, which only includes cortical ROIs. Given the important role subcortical regions play on cognitive functions, we also tested the impact of including these subcortical regions in the input connectivity matrix on prediction accuracy for all 3 cognitive outcomes. For this analysis, we generated 61 subcortical and cerebellar ROIs (Hippocampus: n=4; Thalamus: n=12; Cerebellum: n=27; Basal Ganglia: n=16; Amygdala: n=2) using previously published MNI coordinates [12]. These regions were defined using a winner-take-all partitioning technique for network assignments [13]. In-house Matlab scripts were used to generate spherical ROIs around each center coordinate (radius=4 mm). These 61 spherical ROIs were used in combination with the 333 cortical parcels from Gordon et al. parcellation [4]. We used the same predictive modeling pipeline as in our primary analyses. The average prediction accuracy across 100 iterations determined the prediction accuracy for a given cognitive outcome in the HCP-EP sample.

*Misclassification index*

Following published methods [14,15], we utilized a linear support vector machine (SVM) algorithm to classify cognitive scores as low (-1) or high (1). For this analysis, cognitive scores were binarized as low and high for those who scored 1/3 of the standard deviation below and above the sample mean respectively. The average scorers within these cutoff points were removed to minimize ambiguity in cognitive outcomes. We used leave-one-out cross-validation, where one subject was left out at each iteration and used as a test while remaining participants were used as the training set. At each cross-validation iteration, we randomly subsampled the low and high scorer groups in the training set to equalize the class sizes (30 participants in each group). At each iteration, edges were correlated with the cognitive outcome labels and relevant features were selected (p<0.01). Edges in the positive and negative sets were summed up to obtain connectivity strength summary scores. A linear SVM classifier (*fitcsvm* in Matlab) was trained to relate these summary scores to the binary low and high cognitive scores in the training set. The summary scores were then calculated for the test participant using the selected features and the SVM classifier predicted the outcome for the test participant (as low or high). The test participant was considered misclassified if the predicted label did not match the true label for a given cognitive outcome. This procedure was repeated 100 times using distinct, randomly generated subsamples. For each participant, a misclassification index (MI) was computed as the ratio of the number of times the participant was misclassified to number of all 100 iterations (Figure S2).

*Clinical and demographic correlates of misprediction*

To elucidate potential contributing factors to misprediction, we analyzed the association between misclassification index for the total cognition outcome and 8 key clinical and demographic features (i.e., covariates) in early psychosis that were available in the HCP-EP dataset. These covariates included age, sex, race, parental socioeconomic status (SES), antipsychotic exposure (in months), Positive and Negative Syndrome Scale (PANSS) positive symptom score, PANSS negative symptom score, and head motion (measured by framewise displacement during the resting state scan). Following published methods [14], due to the non-normal distribution of misclassification index, we computed Spearman’s rank correlation between misclassification index and the continuous covariates. For the binary covariates (sex, race), we used the Wilcoxon signed rank test. For those covariates depicting nominally significant correlations (or significant difference for binary covariates) with misclassification index (p<0.05), we further examined their relationship to total cognition scores separately for participants with low and high misclassification indices. For those covariates that showed significant associations with MI, we ran our initial models this time using a partial correlation to predict total cognition while covarying for the covariate of interest. The significance of the predictions was determined by the same permutation procedure used in our initial models (N=1000).

*Models specific to correctly classified participants*

To analyze the differences in brain-behavior relationships between correctly and incorrectly classified participants, we built models using only participants who were frequently correctly classified (i.e., those with MI<0.4) and tested these models on frequently misclassified participants (i.e., those with MI>0.6). For this analysis, we utilized the continuous-outcome framework and 100 train-test splits as in our initial models. At each iteration, we created random train (from correctly classified participants) and test (from misclassified participants) subgroups separated based on their misclassification indices. As in our initial models, the prediction accuracy was computed as the Pearson’s correlation between predicted and observed cognitive scores (p<0.05).

*Analysis of scanner site and site-specific models*

Given previously reported scanner site effects on functional connectivity [16], we performed analyses to assess how well our predictive models generalize between the different sites of the HCP-EP. The HCP-EP cohort used in our analyses were scanned at one of the three sites: Indiana University (N=41), Brigham and Women’s Hospital (N=34), and McLean Hospital (N=17). It is important to note that the cohorts in these sites exhibit differences in certain key demographic and clinical variables. For example, 94% of the participants at the McLean site identified as White, whereas this ratio was 53% for the Brigham site and only 29% for the Indiana site. 59% of the participants at the McLean site had a diagnosis of affective psychosis, while this ratio was 15% for the Indiana site and 24% for the Brigham site. To assess the impact of scanner site on predictions, we first conducted a partial correlation analysis relating connectivity to the total cognition outcome using scanner site as a covariate. To do so, we ran our original total cognition model including scanner site as a covariate and using the same train/test split framework as in our initial models. The Pearson’s correlation between the predicted and observed outcomes determined prediction accuracy at each iteration. The significance of the predictions obtained with this model was determined by the same permutation procedure (N=1000) used in our initial models (p<0.05).

In addition, we built site-specific models using exclusively the fMRI and behavioral data from one of the bigger sites in the HCP-EP (e.g., Indiana University or Brigham and Women’s Hospital) and tested these models on the remaining sites. We utilized a similar train/test split approach as in our original models, creating unique subsets of training and test data at each cross-validation step. We did not build models specific to the small HCP-EP site (i.e., McLean Hospital) due to the very small sample it offers for the training set. At each iteration, 80% of the participants were randomly selected from the training and test sites, with the exception of McLean Hospital (when used as a test site), for which we included the entire site data due to the small sample. All other steps including feature selection, feature summarization and model building mirrored our initial predictive model analyses. The Pearson’s correlation between the observed and predicted outcomes in the test site determined the prediction accuracy for these models. Finally, to determine the extent to which predictive features overlap between our initial (i.e., site-independent) models and site-specific models, we correlated the network summary matrices between site-specific models and the initial (site-independent) models. These summary matrices reflected the fraction of features in a network that are consistently selected across different iterations of the cross-validation.

**Supplementary Results**

*Impact of feature-selection threshold and subcortical features on prediction accuracy*

To examine the effect of using different feature-selection thresholds when building CPMs on prediction accuracy, we ran our initial models in the HCP-EP sample using feature-selection thresholds ranging between p<0.05 and p<0.0001. Table S2 displays prediction accuracy for each threshold and cognitive outcome. Setting more stringent thresholds dramatically reduced the number of input features and led to lower prediction accuracy. For instance, the average number of features selected across all cross-validation iterations for fluid cognition was 1440 at p<0.05, whereas it was 182 at p<0.005, and only 24 at p<0.0005. This suggests that the connectivity strength summary score requires a certain number of features to reach a significant predictive power, over which predictive power plateaus.

We also tested the effect of including subcortical ROIs in the connectivity matrix on prediction accuracy. When using the whole-brain connectivity matrix, prediction accuracy numerically increased for the fluid component of cognition, however, overall did not have a significant impact (p>0.05). Figure S3 shows the distribution of prediction accuracy for each outcome when using only cortico-cortical connectivity vs. whole brain connectivity. Taken together, our findings indicate that the summarized connectivity strength score used in our models appears to be robust to certain changes in input data (e.g., the number of input features, inclusion of subcortical ROIs), however, using stringent thresholds that highly limit the number of features lead to poorer prediction performance.

*Impact of scanner site on predictions and site-specific models*

To examine the impact of scanner site on prediction accuracy, we ran our initial models using scanner site as a covariate. Although the prediction accuracy for total cognition decreased from 0.30 to 0.22 after covarying for scanner site, the predictions remained significant (p<0.05). Additionally, we built site-specific models using data exclusively from one of the two sites with larger samples. The performance of these models is comparable to the original models (r values ranging between 0.22-0.36) when tested on the other major site (Table S5). The performance was worse when such models were tested on the smaller McLean site (r values ranging between -0.08-010), however, the models that only utilized negative feature sets produced considerably higher prediction accuracy in the McLean site as well (r values ranging between 0.13-0.32).

Important network features associated with site-specific models largely overlapped with those in the original (i.e., site-independent) models for the larger sites (r range: [0.19 - 0.64]), whereas the overlap was modest for the smaller site (r range: [-0.04 - 0.24]). Both small and large sites showed greater feature overlap with the original models for the fluid cognition (vs. total cognition) outcome (Table S6), indicating the relative robustness of the models representing the fluid subdomain.

**Supplementary Figures**

**
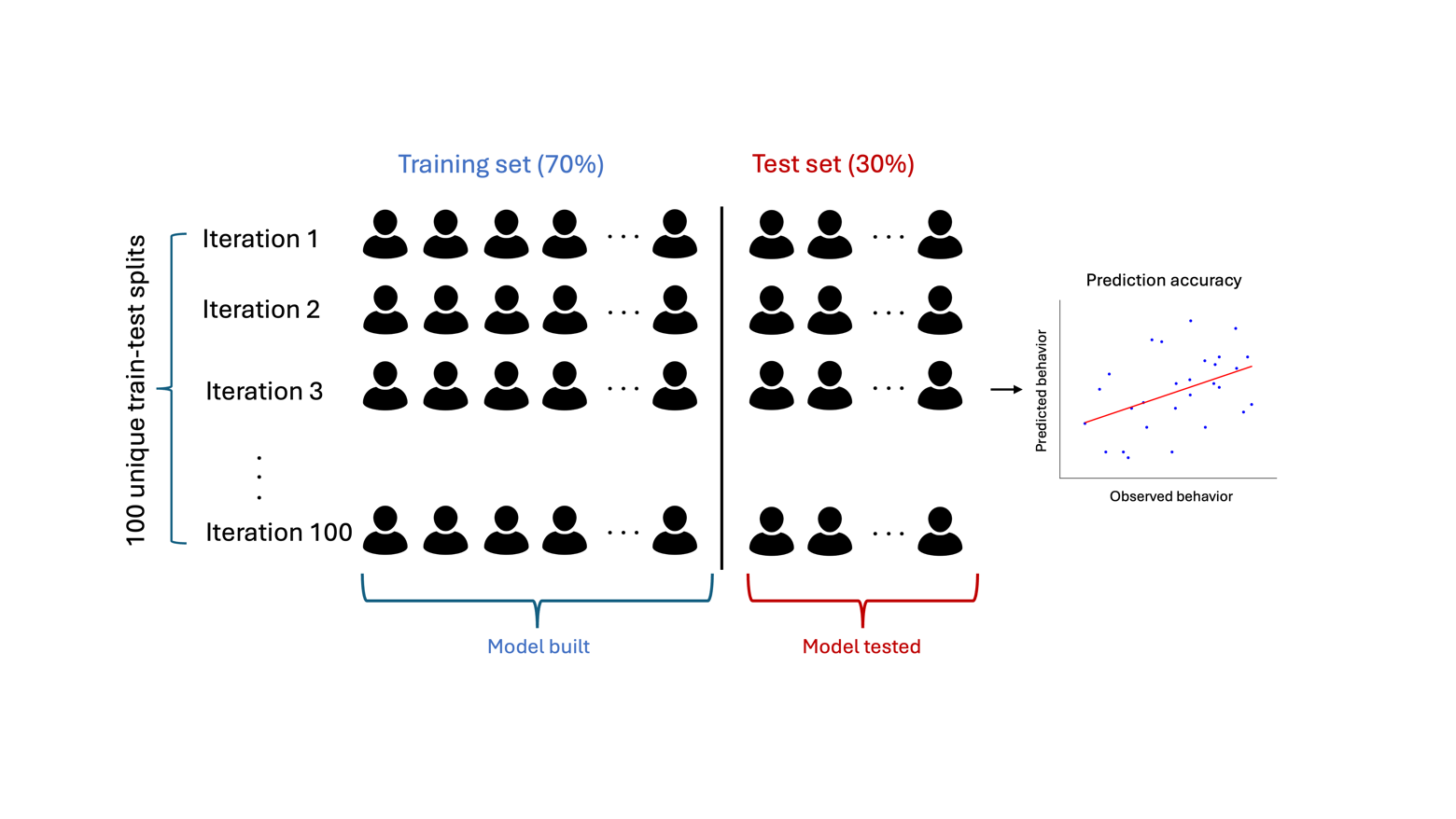
**

**Figure S1.** Schematic illustration of the predictive modeling framework. Unique and random 70%-30% train-test splits were created at 100 iterations. At each iteration, CPMs were built using the training set and tested on the test set. Prediction accuracy was calculated as the Pearson’s correlation between predicted and observed cognitive scores.


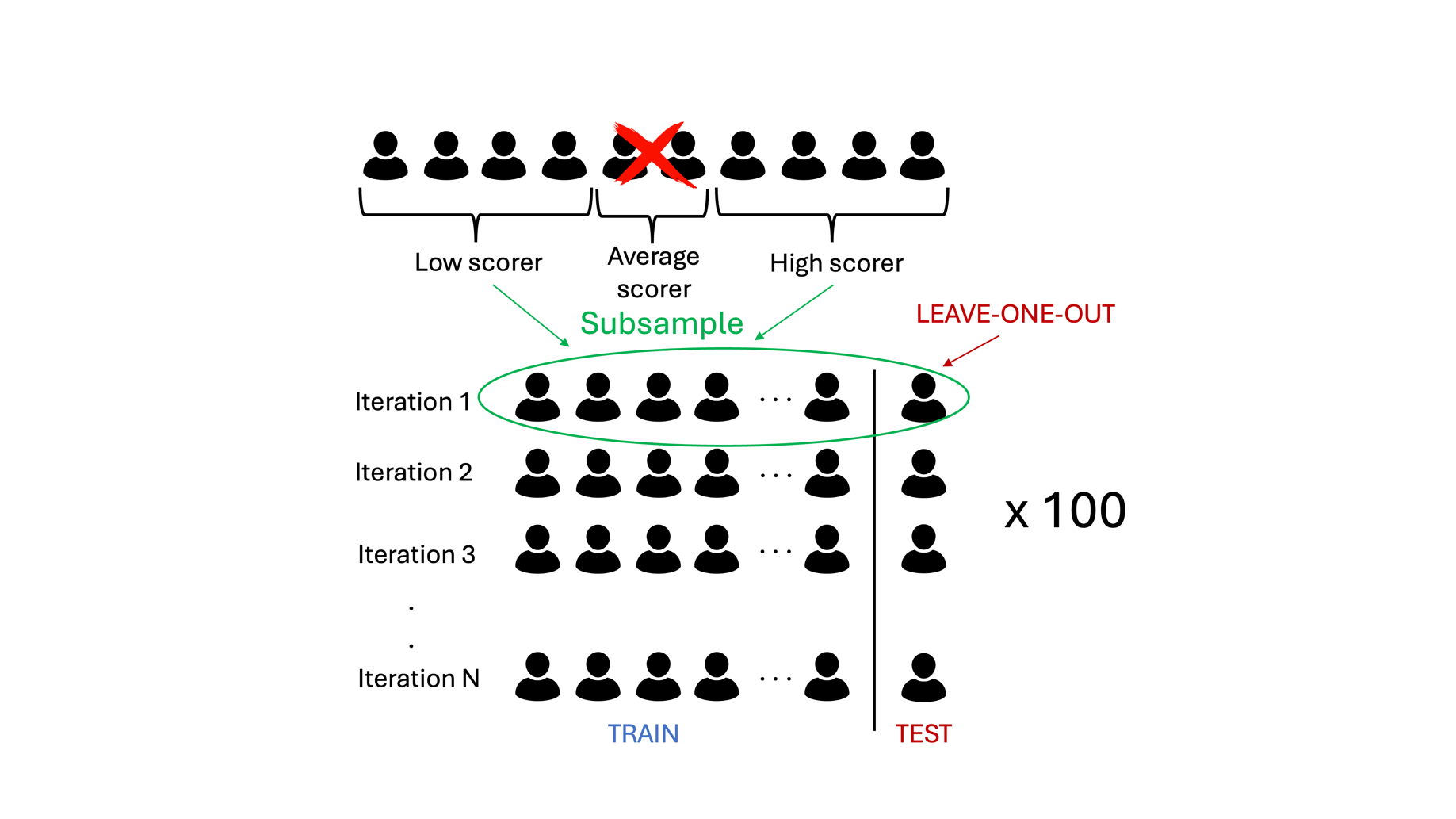


**Figure S2.** Misprediction analysis framework. Leave-one-out cross-validation was performed using an SVM framework to compute misclassification index. Participants were divided into high and low scoring groups, and the average scorers were removed to reduce ambiguity in the assignment. Random subsamples containing equal number of low and high scorers were generated. At each iteration, the SVM model was built on the training set and tested on the left-out participant by predicting the test participant’s performance label (high or low scorer). Each participant in the subsample was used once as a test participant. This procedure was repeated 100 times using distinct, randomly generated subsamples. A participant was considered misclassified if their predicted label did not match their actual label. Misclassification index represents the rate at which a participant was misclassified over 100 iterations.

**Figure S3.** Distribution of prediction accuracy over cross-validation iterations is displayed for each cognitive outcome and for cortical vs. whole brain connectivity features. Including subcortical features in the connectivity matrix numerically increased the prediction accuracy for fluid cognition, however, did not have a significant impact. The box plot on each violin depicts the interquartile range with the center line representing the median, whereas the dots show outliers.


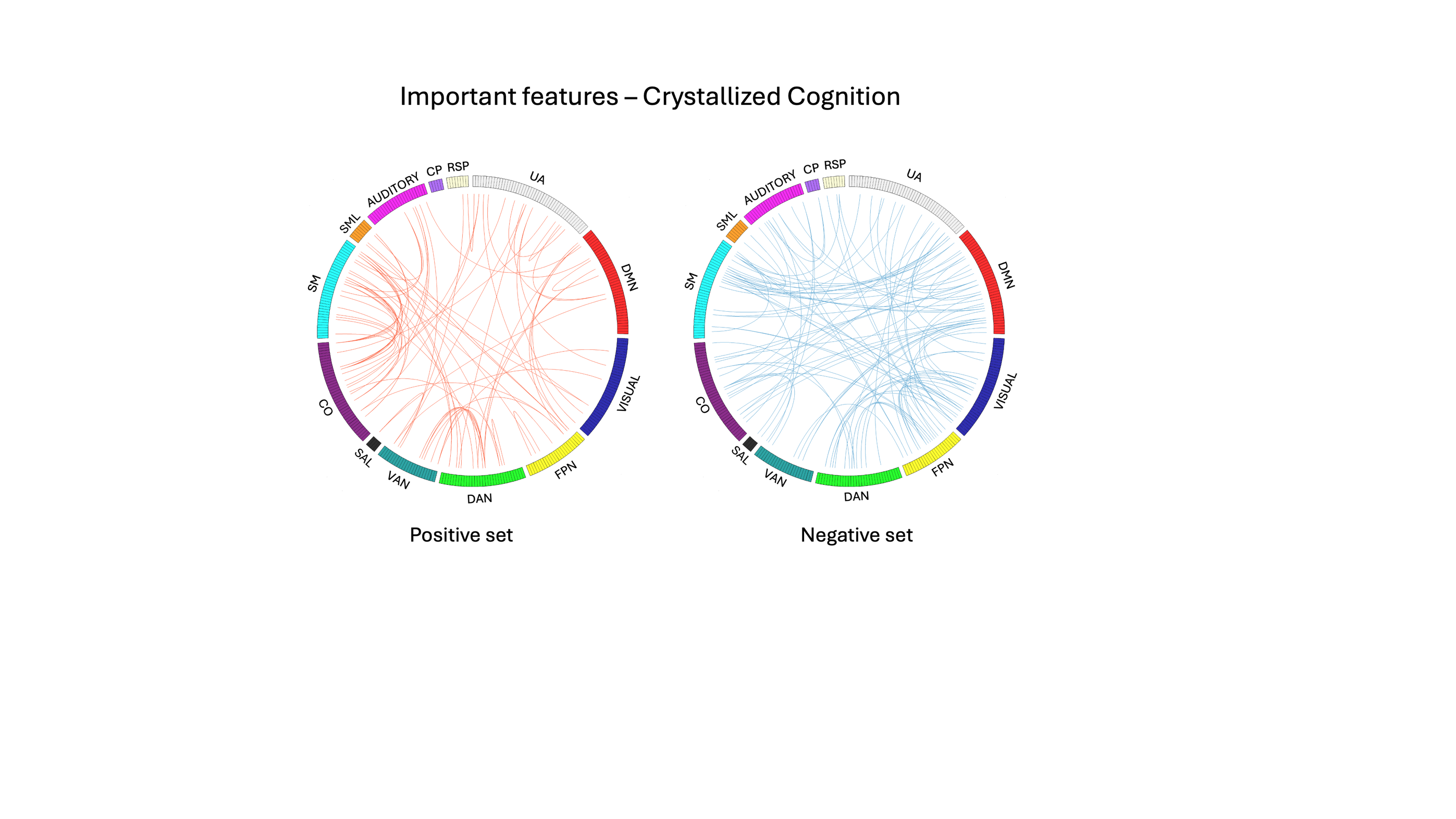


**Figure S4.** Features frequently selected across different cross-validation iterations when predicting crystallized cognition scores. As in fluid cognition, DAN-VAN connectivity was implicated among positive features. Unlike fluid cognition, DMN-VIS connectivity features were not frequently selected in crystallized cognition models. AUD: auditory network, CO: cingulo-opercular network, CP: cingulo-parietal network, DAN: dorsal attention network, DMN: default mode network, FPN: frontoparietal network, RSP: retrosplenial network, SAL: salience network, SM: somatomotor network, SML: lateral somatomotor network, UA: unassigned, VAN: ventral attention network, VIS: visual network.

**
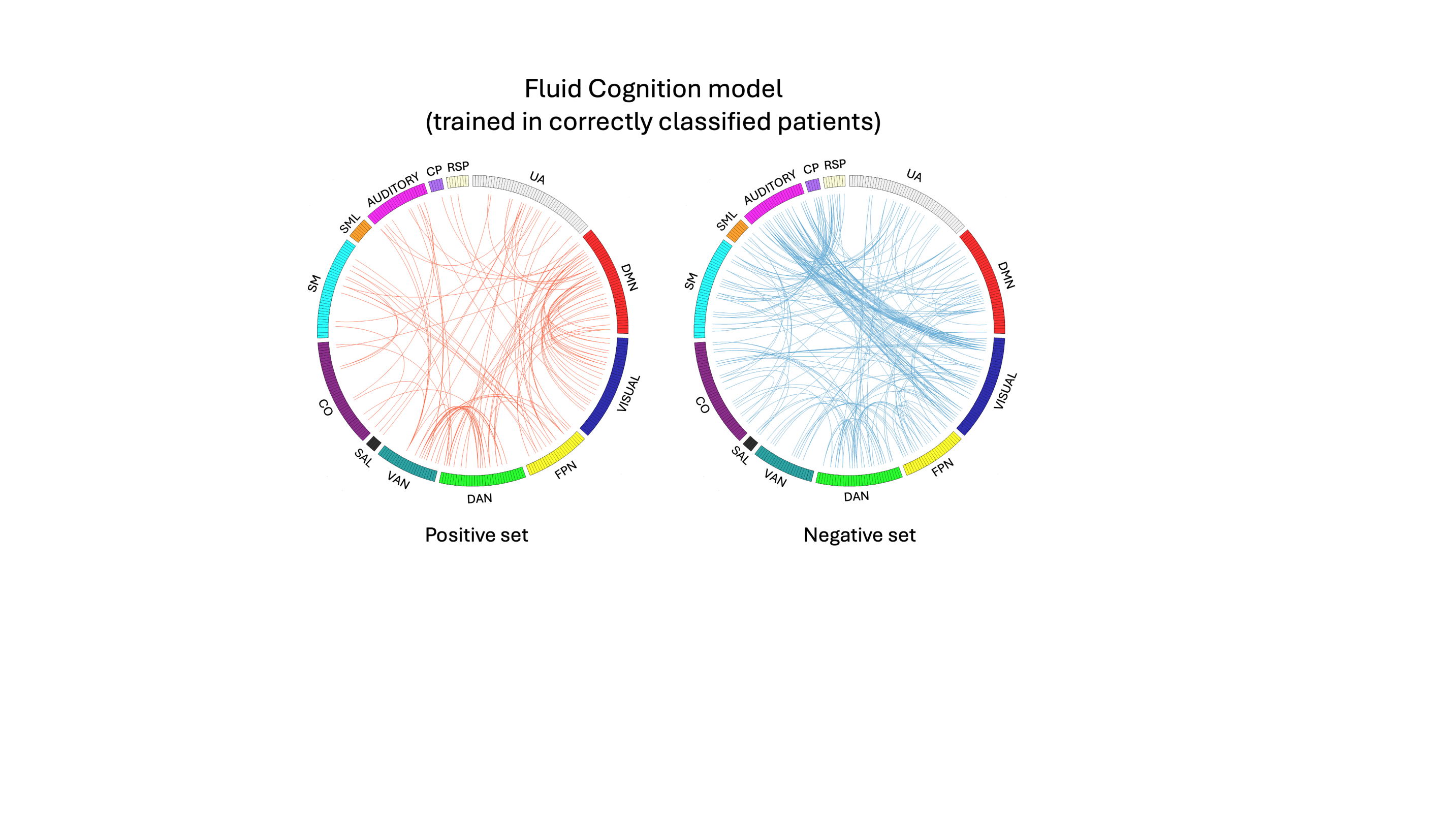
**

**Figure S5.** Important features for predicting fluid cognition in models exclusively trained in frequently correctly classified participants. DMN connectivity with task-positive networks and DAN-VAN connectivity features were frequently selected in the positive feature set. Visual-auditory network edges and somatomotor-retrosplenial network edges were frequently selected in the negative feature set. AUD: auditory network, CO: cingulo-opercular network, CP: cingulo-parietal network, DAN: dorsal attention network, DMN: default mode network, FPN: frontoparietal network, RSP: retrosplenial network, SAL: salience network, SM: somatomotor network, SML: lateral somatomotor network, UA: unassigned, VAN: ventral attention network, VIS: visual network.

**Supplementary Tables**

**Table S1.** Clinical and demographic characteristics of the patients in the HCP-EP and MGH samples.

| **HCP-EP sample** |  |
| --- | --- |
| *Age (years) | 22.91 (3.71) |
| Sex | 36/92 (39.13%) Female |
| Race | 46/92 (50%) White,  36/92 (39.13%) Black or African American, 7/92 (7.61%) Asian, 1/92 (1.09%) American Indian/Alaskan Native |
|  |  |
| Total PANSS Score | 47 (14.69) |
| Age-Adjusted NIH Toolbox Total Cognition Score | 92.12 (19.64) |
| Lifetime Duration of Antipsychotic Exposure (months) | 18.73 (15.72) |
| **CPZE (mg) | 380.55 (206.42) |
| **MGH sample** |  |
|  |  |
| Age | 24.73 (3.45) |
| Sex | 8/20 (40%) Female |
| Race | 10/20 (50%) White,  5/20 (25%) Asian, 2/20 (10%) Black or African American, 3/20 (15%) Mixed Race |
|  |  |
| Brief Psychiatric Rating Scale (BPRS) Score | 30.74 (8.48) |
| Time Since Psychosis Onset (years) | 3.94 (1.71) |
| *Mean and standard deviation values are depicted for quantitative variables. CPZE: Chlorpromazine equivalent dose. **CPZE data were available only for a subset of patients (N=36) in the HCP-EP sample. | |

**Table S2.** The impact of feature-selection threshold on prediction accuracy.

| **Threshold** | **Total** | **Fluid** | **Crystallized** |
| --- | --- | --- | --- |
| p<0.05 | 0.31 | 0.31 | 0.25 |
| p<0.01 | 0.30 | 0.29 | 0.22 |
| p<0.005 | 0.29 | 0.28 | 0.22 |
| p<0.001 | 0.28 | 0.21 | 0.14 |
| p<0.0005 | 0.26 | 0.19 | 0.16 |
| p<0.0001 | 0.16 | 0.12 | 0.12 |
| Mean prediction accuracy is displayed for each cognitive outcome in the HCP-EP sample for different thresholds. Prediction accuracy is calculated via Pearson’s correlation between predicted and observed cognitive scores at each cross-validation iteration. | | | |

**Table S3.** The effect of virtual lesioning of networks on prediction accuracy.

| **Lesioned network** | **TOTAL** | | **FLUID** | |
| --- | --- | --- | --- | --- |
|  | Positive set | Negative set | Positive set | Negative set |
| DMN | 0.015 (0.013) | -0.036 (0.014) | **-0.115 (0.015)**** | -0.017 (0.014) |
| VIS | 0.022 (0.013) | -0.006 (0.013) | -0.032 (0.014) | -0.015 (0.015) |
| FPN | -0.055 (0.014)* | 0.006 (0.015) | -0.053 (0.017)* | 0.005 (0.014) |
| DAN | -0.015 (0.015) | -0.037 (0.015) | 0.016 (0.016) | -0.020 (0.015) |
| VAN | -0.046 (0.016)* | 0.004 (0.015) | -0.025 (0.015) | -0.031 (0.013) |
| SAL | 0.004 (0.013) | 0.032 (0.014) | -0.028 (0.015) | -0.008 (0.012) |
| CON | -0.018 (0.014) | 0.019 (0.013) | 0.020 (0.014) | -0.003 (0.014) |
| SM | -0.014 (0.013) | **-0.088 (0.014)**** | 0.001 (0.015) | -0.039 (0.013) |
| SML | 0.006 (0.014) | 0.018 (0.013) | 0.028 (0.015) | -0.009 (0.013) |
| AUD | 0.034 (0.014) | 0.019 (0.013) | 0.025 (0.013) | -0.003 (0.013) |
| CP | 0.004 (0.013) | -0.017 (0.013) | 0.014 (0.014) | -0.019 (0.015) |
| RSP | 0.031 (0.014) | -0.032 (0.016) | -0.035 (0.017)* | **-0.06 (0.015)**** |
| Mean (Std Dev) of change in prediction accuracy after lesioning a given network is displayed. Negative values indicate a decrease in prediction accuracy, whereas positive values indicate increases. Values with single asterisks depict nominally significant changes in prediction accuracy (p<0.05). Boldfaced values with double asterisks depict significant changes after correction for multiple comparisons (p<0.05 corrected). AUD: auditory network, CO: cingulo-opercular network, CP: cingulo-parietal network, DAN: dorsal attention network, DMN: default mode network, FPN: frontoparietal network, RSP: retrosplenial network, SAL: salience network, SM: somatomotor network, SML: lateral somatomotor network, UA: unassigned, VAN: ventral attention network, VIS: visual network. | | | | |

**Table S4.** Relationships between all covariates and misclassification index (MI).

|  | **Age** | **Sex** | **Race** | **SES** | **AP Exposure** | **PANSS positive** | **PANSS negative** | **Head motion** |
| --- | --- | --- | --- | --- | --- | --- | --- | --- |
| **Low scorer** | r=-0.08  p=0.63 | p=0.86 | p=0.12 | r=0.39 p=0.017* | r=-0.17  p=0.33 | r=-0.19  p=0.26 | r=0.06  p=0.73 | r=-0.2  p=0.22 |
| **High scorer** | r=0.07  p=0.69 | p=0.77 | p=0.22 | r=-0.33  p=0.057 | r=0.35  p=0.03* | r=0.03  p=0.86 | r=-0.11  p=0.53 | r=0.02  p=0.91 |

**Table S5.** Prediction accuracy of site-specific models.

|  | **IU model** | |
| --- | --- | --- |
| **Test site** | TOTAL | FLUID |
| Brigham | 0.29 | 0.36 |
| McLean | -0.07 | 0.002 |
| McLean* | 0.13 | 0.18 |
|  | **Brigham model** | |
| **Test site** | TOTAL | FLUID |
| IU | 0.22 | 0.32 |
| McLean | -0.08 | 0.10 |
| McLean* | 0.18 | 0.32 |
| *Prediction accuracy of models built using data from one of the larger sites (IU or Brigham) when tested on the remaining sites is shown. Asterisks (*) denote the models that only use negative feature sets to make predictions. IU: Indiana University.* | | |

**Table S6.** The overlap of predictive features between site-specific and site-independent models.

| Cognitive outcome / feature set | **IU model** | **Brigham model** | **McLean model** |
| --- | --- | --- | --- |
| TOTAL / positive | 0.2 | 0.45 | 0.04 |
| FLUID / positive | 0.64 | 0.53 | 0.24 |
| TOTAL / negative | 0.38 | 0.19 | -0.04 |
| FLUID / negative | 0.59 | 0.4 | 0.19 |
| *Pearson’s correlations of network summary matrices between site-specific and site-independent models are shown. IU: Indiana University* | | | |

**Supplementary References**

1 Lewandowski KE, Bouix S, Ongur D, Shenton ME. Neuroprogression across the Early Course of Psychosis. J Psychiatr Brain Sci. 2020;5.

2 Jacobs GR, Coleman MJ, Lewandowski KE, Pasternak O, Cetin-Karayumak S, Mesholam-Gately RI, et al. An Introduction to the Human Connectome Project for Early Psychosis. Schizophr Bull. 2024.

3 Power JD, Mitra A, Laumann TO, Snyder AZ, Schlaggar BL, Petersen SE. Methods to detect, characterize, and remove motion artifact in resting state fMRI. Neuroimage. 2014;84:320-41.

4 Gordon EM, Laumann TO, Adeyemo B, Huckins JF, Kelley WM, Petersen SE. Generation and Evaluation of a Cortical Area Parcellation from Resting-State Correlations. Cereb Cortex. 2016;26(1):288-303.

5 Van Dijk KR, Sabuncu MR, Buckner RL. The influence of head motion on intrinsic functional connectivity MRI. Neuroimage. 2012;59(1):431-8.

6 Power JD. A simple but useful way to assess fMRI scan qualities. Neuroimage. 2017;154:150-58.

7 Weintraub S, Dikmen SS, Heaton RK, Tulsky DS, Zelazo PD, Slotkin J, et al. The cognition battery of the NIH toolbox for assessment of neurological and behavioral function: validation in an adult sample. J Int Neuropsychol Soc. 2014;20(6):567-78.

8 Zelazo PD, Anderson JE, Richler J, Wallner-Allen K, Beaumont JL, Conway KP, et al. NIH Toolbox Cognition Battery (CB): validation of executive function measures in adults. J Int Neuropsychol Soc. 2014;20(6):620-9.

9 Heaton RK, Akshoomoff N, Tulsky D, Mungas D, Weintraub S, Dikmen S, et al. Reliability and validity of composite scores from the NIH Toolbox Cognition Battery in adults. J Int Neuropsychol Soc. 2014;20(6):588-98.

10 Cassetta BD, Menon M, Carrion PB, Pearce H, DeGraaf A, Leonova O, et al. Preliminary examination of the validity of the NIH toolbox cognition battery in treatment-resistant psychosis. Clin Neuropsychol. 2020;34(5):981-1003.

11 Feng C, Yuan J, Geng H, Gu R, Zhou H, Wu X, et al. Individualized prediction of trait narcissism from whole-brain resting-state functional connectivity. Hum Brain Mapp. 2018;39(9):3701-12.

12 Seitzman BA, Gratton C, Marek S, Raut RV, Dosenbach NUF, Schlaggar BL, et al. A set of functionally-defined brain regions with improved representation of the subcortex and cerebellum. Neuroimage. 2020;206:116290.

13 Greene DJ, Laumann TO, Dubis JW, Ihnen SK, Neta M, Power JD, et al. Developmental changes in the organization of functional connections between the basal ganglia and cerebral cortex. J Neurosci. 2014;34(17):5842-54.

14 Greene AS, Shen X, Noble S, Horien C, Hahn CA, Arora J, et al. Brain-phenotype models fail for individuals who defy sample stereotypes. Nature. 2022;609(7925):109-18.

15 Eryilmaz H, Dowling KF, Hughes DE, Rodriguez-Thompson A, Tanner A, Huntington C, et al. Working memory load-dependent changes in cortical network connectivity estimated by machine learning. Neuroimage. 2020;217:116895.

16 Yu M, Linn KA, Cook PA, Phillips ML, McInnis M, Fava M, et al. Statistical harmonization corrects site effects in functional connectivity measurements from multi-site fMRI data. Hum Brain Mapp. 2018;39(11):4213-27.
